# Fast Optical Humidity Sensor Based on Hydrogel Thin Film Expansion for Harsh Environment †

**DOI:** 10.3390/s19050999

**Published:** 2019-02-26

**Authors:** Anton Buchberger, Sebastian Peterka, Anna Maria Coclite, Alexander Bergmann

**Affiliations:** 1 Institute of Electronic Sensor Systems, Graz University of Technology, 8010 Graz, Austria; alexander.bergmann@tugraz.at; 2 Institute of Solid State Physics, NAWI Graz, Graz University of Technology, 8010 Graz, Austria; sebastian.peterka@tugraz.at (S.P.); anna.coclite@tugraz.at (A.M.C.)

**Keywords:** humidity measurement, polymer, hydrogel, thin film, initial chemical vapor deposition, laser interference, spectral reflectance, Flory–Huggins theory, interaction parameter

## Abstract

With the application of a recently developed deposition method called initiated chemical vapor deposition (iCVD), responsive hydrogel thin films in the order of a few hundred nanometers were created. When in contact with humid air, the hydrogel layer increases its thickness considerably. The measurement of the thickness change was realized interferometrically with a laser and a broadband light source in two different implementations. The relative change in thickness with respect to humidity can be described with the Flory–Huggins theory. The required Flory–Huggins interaction parameter was determined for the actual hydrogel composition. The setup was designed without electric components in the vicinity of the active sensor layer and is therefore applicable in harsh environments such as explosive or corrosive ones. The implemented sensor prototype delivered reproducible relative humidity (RH) values and the achieved response time for an abrupt change of the humidity $\tau_{63}\leq 2.5$ s was about three times faster compared to one of the fastest commercially available sensors on the market.

## 1. Introduction

Water is involved in many processes in life and technology and plays an important role in the climate system. The term moisture refers to the amount of liquid water in a gas in the form of small droplets, whereas by humidity the amount of gaseous water is meant [1,2]. Humidity is connected to the likelihood for phenomena such as precipitation, dew or fog to happen. Certainly, humidity measurements are a substantial source of information for meteorologists, but there are several other fields in science and industry, where they play an important role as well, ranging from process monitoring to medical investigations [3,4].

Devices for measuring humidity are called hygrometers, which quantify the amount of water vapor in the air. There are several principles ranging from ancient methods based on the elongation of a hygroscopic material (e.g., hair-tension hygrometer), measuring the temperature difference between a dry and wet bulb (psychrometer) to optical detection of the condensation on a chilled surface (dew point hygrometer) [5,6]. A very accurate method is the gravimetric determination of the amount of water, which is quite time consuming and therefore mostly used for standards and calibration purposes [7]. However, the dominant technology nowadays is an electric measurement of humidity [2]. Most sensor devices are based on the change of the dielectric constant or the impedance of a polymer or metal oxide thin film layer and are well suited for most applications [8,9]. Those sensors provide typical response times in the order of a few seconds [2]. There are several fields where faster and more robust sensors are necessary, such as breath recording or monitoring gas-flows [4,10]. In most of the above mentioned examples of hygrometers, the sensitive property is proportional to the total mass of water in the air, which is called the absolute humidity. A more commonly used quantity is the percentage relative humidity RH. It is defined as the ratio of the partial pressure of water vapor in the air, which is related to the total mass of water, over the equilibrium vapor pressure of water [11]. Since the equilibrium vapor pressure is temperature dependent, the relative humidity RH is a temperature dependent quantity. Thus, measuring a property that depends on the absolute humidity always requires an additional measurement of the temperature for the determination of RH.

New materials and measurement techniques are under investigation for the development of novel humidity sensors. A promising class of materials are hydrophilic polymer networks that can absorb water in their meshes, the so-called hydrogels. Electronic properties of these hydrogels are related to the humidity of the surrounding atmosphere [9], but there are other properties depending on it too. In the former work, it was shown that Poly 2-hydroxyethyl methacrylate (pHEMA) thin films, deposited with a method called initiated chemical vapor deposition (iCVD), provide excellent swelling behavior [12]. To achieve a large response in terms of swelling upon exposure to water environment, the polymer thin film must have the right chemistry, i.e., a large quantity of groups that would H-bond water. pHEMA has hydroxyl groups, thus is an ideal candidate for experiments in water. The geometry is also important for achieving large response. Nanofilms have shown faster swelling than bulk hydrogels [13]. The iCVD method allows obtaining polymer thin films, with thickness as low as few nm, of polymers with high chemical fidelity.

The correlation of the relative thickness change and the relative humidity RH is described by the Flory–Huggins theory, which applies for the kind of hydrogel that was used [14,15]. The presented hygrometer is a first principle measurement system that is based on an optical measurement of the swelling of a hydrogel thin-film, which is directly proportional to the relative humidity of the surrounding atmosphere. A sensor based on this principle is capable of measuring the relative humidity directly. The sensor is also sensitive to moisture, thus a non-condensing atmosphere is required. Another advantage of an optical based sensor is the possibility of an application in harsh environments, such as corrosive or even explosive ones, due to the lack of electric and electronic components in the active sensor area. Additionally, the focus of the sensor development is on industrial applicability in terms of costs, dimensions and the denial of ionizing radiation. Therefore, popular thin-film measurement techniques such as ellipsometry or X-ray diffraction (XRD) were not taken into account.

## 2. Materials and Methods

Developing a new sensor principle involves contributions from different fields of science. Certainly an important part is the hydrogel itself and its deposition, which is well-explored in the field of polymer, surface and thin-film physics [16,17,18,19,20]. Furthermore, the deposition has to be adapted to the optical setup in terms of possible substrate materials and geometry. For reasons of simplicity and availability, it was decided to realize the setup in the visible range. To apply the sensor also in harsh environment, no electrical parts for the optical detection were placed on the side of the hydrogel thin-film. This means that the light sources as well as the components for optical detection were installed on the backside of the substrate in a reflective alignment. The captured data were evaluated with self developed algorithms in MATLAB.

### 2.1. Flory–Huggins Theory

Variation of RH in the atmosphere that surrounds a hydrogel thin film leads to a change of its thickness, according to former experiments. The correlation between those quantities is described with the Flory–Huggins theory by Equation (1) [12].

(1) $$RH=\left(1-\frac{d_{0}}{d}\right)\exp\left(\frac{d_{0}}{d}+\chi {\left(\frac{d_{0}}{d}\right)}^{2}\right)$$

It contains just the thickness ratio $d_{0}/d$ and does not require a measurement of an absolute thickness value. A measurement method that delivers just relative thickness changes would be sufficient.

Furthermore, Equation (1) contains the so-called Flory–Huggins interaction parameter χ, which is non-constant and specific for the hydrogel–solvent combination used and can display non-trivial dependencies on hydrogel composition, chain length and temperature [14]. An analytical expression in terms of material parameters cannot be found for it. Hence, it must be empirically approximated for the actual hydrogel composition by a measurement of the thickness variation and the corresponding RH value simultaneously. With the derived χ and Equation (1), the corresponding RH can be calculated for every thickness value measured with the same hydrogel composition and conditions.

### 2.2. Hydrogel Thin Film

The iCVD method allows obtaining polymer thin films, with thickness as low as few nm, with high chemical fidelity, and conformally covering patterned substrates. It is based on the mechanism of radical polymerizations and takes place completely under medium vacuum conditions. The radicals are created by thermal fragmentation of a peroxide (tert-butyl peroxide, TBPO, Sigma-Aldrich, St. Louis, MO, USA) from a hot wire suspended above the substrate. The created radicals react with the vinyl bonds of the monomers, which are vaporized and fed into the vacuum chamber. The substrate is kept at temperatures between 10 and 60 ${}^{\circ }C$, to favor the absorption of the monomers molecules. In this case, the monomer chosen was 2-hydroxyethylmethacrylate (HEMA, Sigma-Aldrich, St. Louis, MO, USA ). When a radical reacts with a monomer molecule absorbed on the surface, a secondary radical is formed, which then reacts further with other monomer molecules. Termination of the polymer chain happens when two radicals react. The polymerization mechanism of iCVD is explained in more detail in several publications [16,17].

In the present study, the chosen deposition conditions were the following. HEMA was heated up to 75 ${}^{\circ }C$, while TBPO was kept at room temperature. The temperatures of the substrate and the hot wire were held at 30 ${}^{\circ }C$ and 220 ${}^{\circ }C$, respectively. The flow rate of HEMA was set to 0.5 sccm and for TBPO to 1 sccm. Nitrogen was fed into the chamber with 3 sccm which led to a total pressure of 250 m Torr. With these conditions, a pure pHEMA hydrogel thin-film layer was deposited on a 2 mm sapphire substrate with an initial thickness of about 600 nm in a custom-built reactor, as described by Ranacher et al. [20]. Previous publications about pHEMA thin films provide further details about the hydrogel and deposition procedure and show that relative thickness increases in the order of 30% [12]. With the chosen initial thickness, this leads to an expansion that should be resolvable by interferometric methods. The used deposition method can be applied to different kinds of substrate materials [18]. The reflectivity at an interface is dependent on the difference in refractive index. Since the refractive index of bulk pHEMA is about 1.44 [21], a transparent, high index material was chosen to optimize the reflectivity. Sapphire turned out to be the best trade-off in terms of costs.

### 2.3. Optical Setup

**Figure 1:**
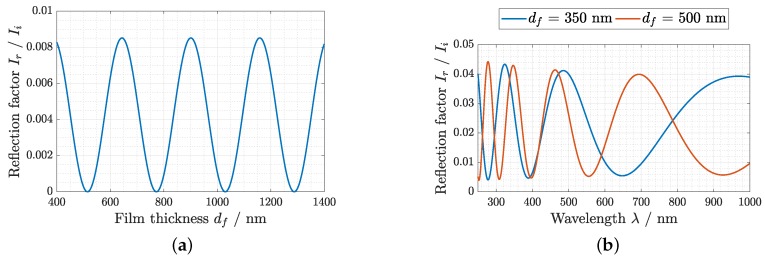
Plots of the analytical model for: (**a**) the laser interference setup for a wavelength of 635 nm at an incident angle of 60.5${}^{\circ }$, which shows the relative reflected intensity over the thin-film thickness; and (**b**) the spectral reflectance at $\theta_{0}=37{}^{\circ }$ for two exemplary thickness values $d_{f}$.

The optical detection was realized in two implementations, which are both based on interference phenomena. The first implementation consists of a laser and a photo detector and is able to measure relative thickness changes with a time resolution of 0.1 s. As long as the total thickness increase is limited to between a maximum and a minimum of the interference curve shown in Figure 1a and the initial thickness at 0% RH is known, the laser interference measurement would be sufficient for the determination of the relative thickness, which is necessary for the Flory–Huggins theory. A higher increase would cause an ambiguous result, which limits the setup to a certain maximum RH value. However, in terms of costs, this is the preferable configuration. The second implementation is realized with a broadband light source and a spectrometer. The time resolution is lower (>3 s), but the spectral recordings yield to absolute thickness values over a broad range causing no restrictions to the applicable RH range.

**Figure 2:**
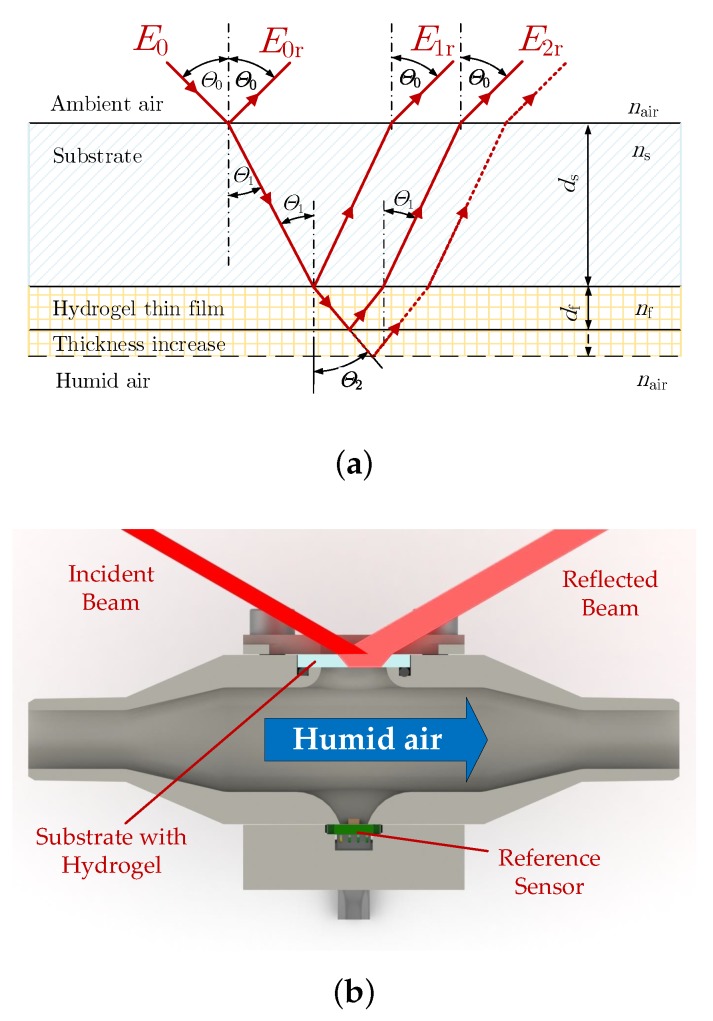
(**a**) Principal sketch of a field that gets reflected at a swelling hydrogel thin-film layer deposited on a substrate (thickness of the hydrogel is drawn exaggerated); and (**b**) sectional view of the practical implementation of the substrate with the hydrogel (drawn in light blue) at the measurement chamber. Reference sensor (in green) on the opposite side.

The optical path and the principal detection setup are sketched in Figure 2. Polarizers were used in order to p-polarize the incident beam. Hence, just p-polarized light is considered for the model. The incoming field $E_{0}$ gets reflected at every interface, leading to three reflected fields that interfere with each other. Higher order reflections can be neglected, because the reflection coefficients are low according to a small difference in refractive index of the hydrogel and the substrate. The interference pattern is described by the total reflected intensity $I_{r}$, which is derived by Equation (2).

(2) $$\begin{array}{cc} I_{r} & =|E_{0r}+E_{1r}+E_{2r}{|}^{2}\\ E_{nr} & =R_{n}E_{i}e^{i(kz-\omega t+\delta_{n})}\;\mathrm{with}\;n=0,1,2 \end{array}$$

where $I_{r}$ is the absolute squared of the sum of all three complex electric fields $E_{nr}$, with a prefactor $R_{n}$ depending on the corresponding reflection and transmission coefficients for p-polarized light and a phase factor $\delta_{n}$. The thin film thickness $d_{f}$ is contained in the phase factor $\delta_{2}$ of the electric field, which is reflected at the interface between the hydrogel and the humid air, described by Equation (3) using the incident angle $\theta_{1}$ and the refractive indices $n_{s}$ and $n_{f}$, of the substrate and the hydrogel, respectively.

(3) $$\delta_{2}=\frac{4\pi d_{f}}{\lambda }\sqrt{n_{s}^{2}-n_{f}^{2}\sin^{2}\theta_{1}}$$

If the wavelength λ of the incoming light is kept constant, as is the case for the laser interference setup, the elaboration of Equation (2) leads to an interference pattern, which is dependent on the thin film thickness. The application of a broadband light source leads to a wavelength dependent interference pattern that is unique for every single thickness value. Therefore, absolute thin film thicknesses can be derived by fitting the model function to recorded reflectance spectra. Initial measurements suggest that the refractive index of the hydrogel $n_{f}$ decreases at very high water uptake. However, this effect is small enough that $n_{f}$ can be assumed to stay constant over the applied humidity range. Similarly, at very high humidity levels (RH>90%), the uncertainty of the thickness evaluation is increased due to small deviations from the idealized model. The corresponding plots of the analytical model for the two different methods are shown in Figure 1. Both measurement principles were implemented such that the data could be recorded simultaneously, by setting up the components at different incident angles.

The experimental setup was implemented with the substrate mounted on the sidewall of a measurement chamber shown in Figure 2b. A Sensirion SHT31 (Sensirion AG, Stäfa, Switzerland) humidity sensor was installed as a reference on the opposite wall. The atmosphere inside the chamber was controlled by a direct evaporator aSTEAM DV-4 series (aDROP Feuchtemeßtechnik GmbH, Fürth, Germany), which was attached with a hose. The temperature was held constant at 24 ${}^{\circ }C$ for all measurements. For the laser interference setup, a Thorlabs CPS635 laser diode and a Thorlabs S120C (Thorlabs Inc., Newton, NJ, USA) photo diode were used. The spectral reflectance measurement was implemented with an Ocean Optics HL-2000-HP-FHSA tungsten halogen light source and an Ocean Optics Flame (Ocean Optics Inc., Winter Parks, FL, USA) UV-VIS spectrometer applied with multi-mode fibers. For compensation of the light source spectrum and wavelength specific absorption of the fibers, all recorded spectra were related to a reference measurement with a sapphire substrate without hydrogel layer. The data for both measurement methods were recorded simultaneously. In parallel, the installed humidity sensor captured a reference RH value.

In another implementation of the optical setup, the hydrogel was deposited onto the tip of a fiber probe, which was illuminated and detected with the same components used for the tabletop solution connected with a fiber beam splitter. This setup was more robust in terms of optical misalignment and therefore easy applicable for future field measurements. The optical path can be described with the same model (Figure 2a) and an incident angle $\theta_{0}=0{}^{\circ }$.

## 3. Results and Discussion

The basic outcomes of the performed measurements were different intensities and raw spectra, which vary with the hydrogel thickness depending on the humidity in the chamber. To evaluate the model shown in Figure 1, the measured raw data were related to thickness values. For the realization of a humidity sensor, the derived hydrogel thickness must be related to the corresponding relative humidity. Therefore, the analytical model by Flory–Huggins was evaluated initially and the Flory–Huggins interaction parameter χ was derived by calibration measurements for the actual material combination of pHEMA and water. Finally, it was possible to determine the relative humidity in the chamber with the hydrogel sensor.

### 3.1. Thickness Measurement

**Figure 3:**
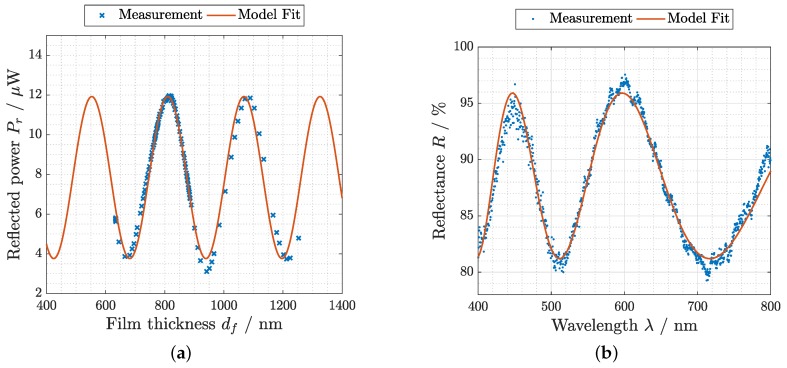
Measurements with corresponding model fit for (**a**) the laser interference setup for a wavelength of 635 nm at an incident angle of 60.5${}^{\circ }$, which shows the reflected power over the thin-film thickness derived by spectral reflectance measurements such as the one shown in (**b**) for an exemplary thickness value of 644.8±0.8 nm at an incident angle of 37${}^{\circ }$. Note: All reflectance spectra are related to a reference measurement with a sapphire substrate without hydrogel layer.

Figure 3b shows a measured reflectance spectrum for an exemplary RH setting and thin-film thickness, respectively. By fitting the data with the analytical model, an absolute thickness was derived. This was performed for several humidity settings between 3 and 97% RH. In Figure 3a, the measured reflected laser power is plotted over the derived absolute thickness values. The fit with the analytical model was in good agreement with the measured data. At film thicknesses >1000 nm the data started to deviate from the model function. This could be caused by a variation of the refractive index of the hydrogel at high humidity levels, which are not taken into account by the model used.

The total increase of the hydrogel thickness can be observed in Figure 3a. It shows that the thickness doubled within the applied humidity range from 3% RH to 97% RH. The applicability of the actually implemented laser interference setup for humidity measurements was limited to 50% RH. Above that humidity level, the thickness of the hydrogel thin-film surpassed 680 nm, at which the model was not monotonic anymore and the result in terms of the corresponding thin film thickness was ambiguous. Note that RH and thickness are exponentially related to each other (Equation (1)), which means that most of the total thickness increase happens in the high RH region. An optimized initial thickness (slightly larger than an extremum in Figure 3a) or lasers with a higher wavelength as well as materials with lower total swelling would extend the applicable RH range. In terms of costs and simplicity of the necessary components, it would be desirable to optimize this configuration. Nevertheless, the results from the spectral reflectance measurements that employ more sophisticated components might be used over the whole applied RH range.

### 3.2. Evaluation Flory–Huggins Theory

**Figure 4:**
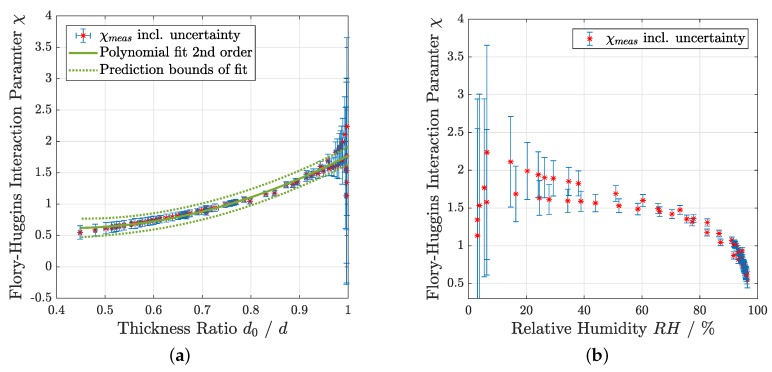
Flory–Huggins interaction parameter χ derived by humidity and thickness measurements as a function of the hydrogel thickness ratio $d_{0}/d$ with corresponding model fit in (**a**) and as a function of the applied relative humidity RH in (**b**). Result for the fit parameters: $\chi_{0}=1.6\pm 0.4$, $\chi_{1}=-4.0\pm 1.0$, and $\chi_{2}=4.2\pm 0.7$.

The Flory–Huggins theory was evaluated over a humidity range from 3% RH to 97% RH. Thus, the data from the spectral reflectance measurements were used for this evaluation. The initial thickness $d_{0}$ at 0% RH that is necessary for the Flory–Huggins model (Equation (1)) was derived through extrapolation of the measured values. Information in the literature about the Flory–Huggins interaction parameter for the used material combination of a pHEMA thin film produced by iCVD and water is limited to a narrow humidity range. Since reference RH values were recorded simultaneously with the hydrogel thickness, χ can be expressed as a function of $d_{0}/d$ by a transformed version of Equation (1) for the whole applied humidity range. According to the literature, χ is expanded by a second-order polynomial in terms of $d_{0}/d$. These parameters were derived by a fit presented in Figure 4a [22].

Figure 4b shows another important observation during the measurements, namely that χ dropped to about 0.5 at 97% RH. Theoretic models show that for good solvents the value for the interaction parameter χ must be less than 0.5, which denotes an approximate limit for dissolution. For values slightly larger than 0.5, it is expected that the polymer is swollen by the solvent [23]. The smallest value of χ that could be experimentally observed was 0.55±0.11 at a maximum relative humidity of 97% RH. Above this RH level, the hydrogel started to dissolve as predicted. However, a hygrometer that starts dissolving at high humidity would be rather impractical. Previous experiments have shown that the hydrogel can be stabilized against liquid water by addition of crosslinkers during the deposition process [12]. Such crosslinked hydrogels would solve this issue, but for the actual experiment a pure pHEMA film was used, because the swelling was demonstrated to be higher. Figure 4 presents values for the Flory–Huggins interaction parameter χ for pHEMA over a broad humidity range at room temperature for the first time. The result is in good agreement with previous measurements using the same materials but over a smaller humidity range and experiments using solution-synthesized pHEMA hydrogels at 35 ${}^{\circ }C$ that report χ values between 1.5 and 1 for RH from 20% to 90% [12,24]. In polymer physics, the interaction parameter is commonly expressed as a function of the volume fraction of the polymer–solvent combination [23]. Since the lateral expansion of a thin film is negligible, the volume fraction is equivalent to the thickness ratio $d_{0}/d$ of the dry hydrogel to the swollen hydrogel. The empirically determined Flory–Huggins interaction parameter for pHEMA and water as the solvent changes from 0.55 to 1.8 over a range of the volume fraction from 0.45 to 1. The error bars were determined by standard propagation of uncertainty. The high uncertainty at low RH (correspondingly $d_{0}/d$ close to 1) was caused by the weak response of the hydrogel thickness in this region, whereas the uncertainty of the thickness values derived by the fit of the spectral reflectance measurements stay constant.

**Figure 5:**
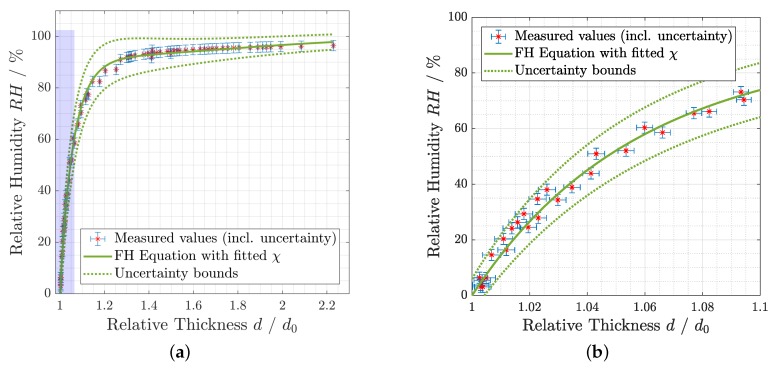
RH calculated with Flory–Huggins theory (Equation (1)) and the empirically determined Flory–Huggins parameter χ for a pHEMA thin film, compared with measured values. For a better visibility, (**b**) is the enlarged version of the blue shaded rectangle in (**a**).

By applying the derived χ in Equation (1), the corresponding RH can be calculated for every thickness value with the same hydrogel composition and conditions, which is plotted in Figure 5. It shows that it is possible to describe the correlation of the measured values with the Flory–Huggins theory satisfyingly with an uncertainty in the order ±10%, which is acceptable considering the early stage of the sensor development. A more accurate thickness measurement and an increase of the response at low RH values would decrease the uncertainty further.

### 3.3. Humidity Measurements

**Figure 6:**
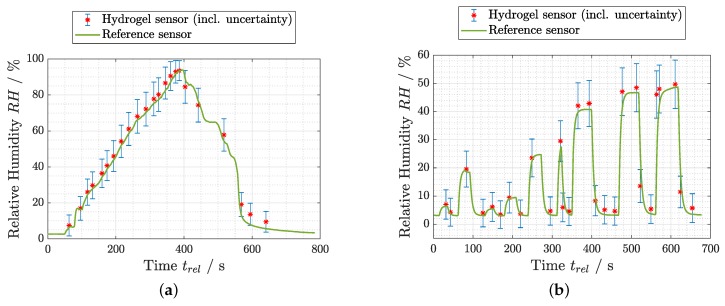
Evaluation measurements of the hydrogel sensor compared to the reference sensor for (**a**) a ramp-like and (**b**) a dynamic change of the humidity RH in the chamber.

**Figure 7:**
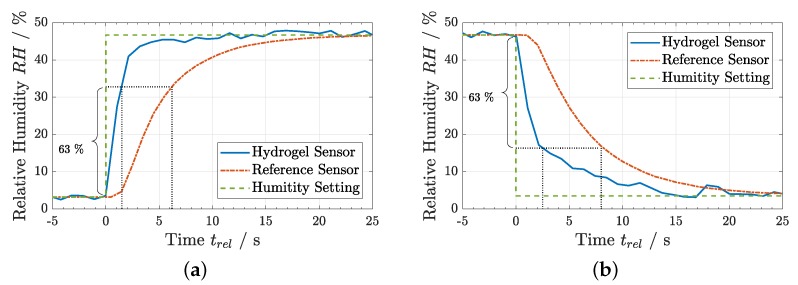
Time response of RH measured with the hydrogel sensor compared to a commercial reference sensor after an abrupt change of the humidity setting for: (**a**) a humidity rise; and (**b**) a humidity drop. The dotted lines indicate the response time parameter $\tau_{63}$.

With the approval of the Flory–Huggins model function and the determination of the corresponding interaction parameter χ for the hydrogel used, evaluation measurements could be performed. Two examples are presented in Figure 6. The plots compare the continuously measured reference RH value with the RH value derived by hydrogel thickness measurements using the spectral reflectance setup at certain times. The error bars were calculated by standard propagation of uncertainty. The hydrogel sensor delivered reproducible RH values compared to the reference sensor in a ramp measurement (Figure 6a) as well as in a dynamic step wise change of the humidity setting (Figure 6b). The latter allows already vague assumptions about the fast response of the hydrogel sensor. For relative humidity values <50% RH, the data from the laser interference setup could also be used, as elaborated in Section 3.1. This method allowed much higher time resolution, which is necessary for a closer investigation of the sensor response time. An abrupt increase and decrease of the humidity level inside the chamber was applied and the response of both sensors analyzed, as shown in Figure 7.

The response time is commonly defined as the 1/e time constant $\tau_{63}$, at which the sensor readings change 63% of the full RH step. The measured response times of the hydrogel sensor were $\tau_{63}=1.5\pm 0.5$ s for an abrupt signal rise and $\tau_{63}=2.5\pm 0.5$ s for an abrupt signal drop. With the reference sensor, response times between 6 and 8 s were achieved, which conformed to its specifications and was at the lower limit achievable with existing electronic sensors [2]. Thus, the hydrogel reacted about three times more quickly than the reference sensor, which is already one of the fastest in the consumer market. In comparison with the humidity decrease, the response time for the increase was about 20–40% lower. This was in agreement with a comparable effect that was observed for thermo-responsive polymers, where the deswelling happened with a larger time scale than the swelling process [25].

The response times of the hydrogel sensor are much shorter than expected from previous experiments [12]. It was assumed that the limiting factor is the thin film thickness, meaning that thinner films would lead to response times even below one second. In addition, an increase of the hydrogel surface, which could be realized by nano-structuring, is expected to reduce the response times further. Such fast sensors would enable an application for high dynamic processes such as breath monitoring in medical research.

## 4. Conclusions

In the presented work, hydrogel thin films produced by initiated chemical vapor deposition were characterized and their capability as humidity sensor was investigated. The theory underlying the sensor by Flory–Huggins was evaluated and the corresponding interaction parameter χ was determined over a broad humidity range for the actual material combination of pHEMA and water. It changes from 1.8 to 0.55 over a humidity range from 97% RH to 3% RH and from 1 to 0.45 expressed in terms of the volume fraction, respectively. Determination of this parameter enabled the application of the hydrogel as a humidity sensor by optical measurement of the thin film thickness. The setup reacts quickly and reproducible to changes of the humidity level. The measured response times are about three times faster compared to one of the fastest commercially available sensors. Two optical detection methods were implemented. The spectral reflectance measurements covered a humidity range from 3 to 97% RH. The application range of the laser interference setup was limited to <50% RH for the actual implementation. Modifications in the composition of the hydrogel and the used laser are expected to increase this range. Due to the lack of electric components in the sensor head, measurements in harsh environments such as corrosive or explosive ones are possible. In a second iteration, the setup was realized with the hydrogel deposited on the tip of a fiber probe, which makes the system more robust concerning optical alignment and very attractive in terms of price. This led to comparable results and is matter of further investigations.
